# Stereological study on the effect of vitamin C in preventing the adverse effects of bisphenol A on rat ovary 

**Published:** 2016-06

**Authors:** Malek Soleimani Mehranjani, Tayebeh Mansoori

**Affiliations:** *Department of Biology, Faculty of Science, Arak University, Arak, Iran.*

**Keywords:** *Ovary*, *Bisphenol A*, *Vitamin C*, *Stereology*, *Rat*

## Abstract

**Background:**

Bisphenol A (BPA), an environmental pollutant, can generate free radicals which damages the reproductive system. Vitamin C is an antioxidant which may prevent the adverse effects of free radicals.

**Objective:**

The aim was to investigate the effect of vitamin C on the ovary tissue in rats treated with BPA.

**Materials and Methods:**

In this experimental study, 24 female Wistar rats (200±20 gr) were randomly divided into 4 groups (n=6): control, BPA (60 µg/Kg/day), vitamin C (150 mg/Kg/day) and BPA + vitamin C and orally treated for 20 days. The left ovaries were taken out, fixed for tissue processing and studied using stereological methods. Data were analyzed with SPSS using one-way ANOVA, and the means were considered significantly different at (p<0.05).

**Results:**

The total volume of ovary and cortex (p<0.01), medulla (p<0.05), the volume of corpus luteum (p<0.001) and the mean number of antral follicles (p<0.001) significantly reduced in BPA group compared with control, while the number of atretic follicles increased (p<0.05). The volume of oocyte (p<0.01) and its nucleus (p<0.001) in the antral follicles and the thickness of zona pellucida (ZP) in the secondary (p<0.05) and antral (p<0.001) follicles significantly decreased in BPA group compared with controls. The above parameters in the BPA + vitamin C group were compensated to control level.

**Conclusion:**

Vitamin C can be used as a potential antioxidant in the case of BPA toxication

## Introduction

Bisphenol A (BPA) is one of the alkali phenols that mimics estrogen and used in the industry for producing epoxy resins, polycarbonate plastics such as plastic food containers, food and beverage can liners, baby bottles, and dental sealants (1) .It is also known as an environmental pollutants (2-4). Due to its estrogenic effects, it can impair the endocrine system causing alterations in sex determination and sex glands performance (5, 6). In rodents, BPA has been shown to affect indices of reproductive functions such as egg deformation, ovary and uterus weights and fertilization rates (7-9). 

BPA also induces oxidative stress through reducing the anti-oxidant enzymes and increasing hydrogen peroxide and lipid peroxidation (10, 11). Fernandez *et al* showed that treatment of female rats with BPA for 10 days postnatal period caused ovary atrophy along with large cysts, an increase in atretic follicles and significant reduction in the number of antral follicles and corpus luteum (12).

Treatment of female rats with 50 mg/kg and 50 µg/kg of BPA for 3 days postnatal period also generated hemorrhagic follicles, big follicles similar to antral follicles, multi-oocyte follicles, and ovary cysts at maturation time (13). In another investigation, treatment of female rats for 6 days postnatal period with BPA leads to ovary cysts formation (14). On the other hand, antioxidants such as vitamin C can prevent the adverse effects of the oxidative stress by inhibiting reactive species of oxygen and nitrogen (15). 

Investigations indicated that co-administration of Vitamin C with other pollutants avoids the harmful effects of these chemicals on female rats reproductive organs. Treatment of adult female rats with vitamin C and sodium arsenite compensated for the reduction in the ovary weight to control level. Vitamin C also reduces lipid peroxidation in rats ovary treated with hexavalent chromium. In another investigation, co-treatment of adult rats with vitamin C and Kmno4 prevented the harmful effects of this chemical on the ovary (16-18).

The aim was to investigate the effect of vitamin C on the rats ovary tissue following BPA exposure using stereological methods.

## Materials and methods

**Animals and treatments**

In this experimental study, 24 adult female Wistar rats with an average weight of 200±20 gr were purchased from Iran Pasteur Institute and kept in Arak University animal house under standard conditions (22±2^o^C and 12 hr light/dark). All experimental procedures were carried out according to the Ethics Committee of Arak University. The rats were then divided into 4 groups: control group, BPA (60 µg/kg/day), BPA + Vitamin C (150 mg/kg/day), and Vitamin C. Oral treatment was carried out by gavage for 20 days (equal to 4 sterous cycles) with a 24 hr interval. Corn oil was used as a carrier for BPA and water was used as a carrier for vitamin C (19, 20). 

**Tissue preparation**

The rats were weighed and anesthetized by diethyl ether at the end of the treatment. The left ovaries were taken out and placed in Bouin fixative for 24 hrs. After tissue processing, the samples were placed in cylindrical paraffin blocks. 

**Stereological study**

Orientator method was used to obtain Isotropic Uniform Random (IUR) sections (21, 22). For this purpose, the cylindrical paraffin block was randomly placed on the φ clock which was divided into 9 equal parts. Then, by choosing a random number from 0 to 9, an appropriate cut was made along the selected number. 

**Figure 1:**
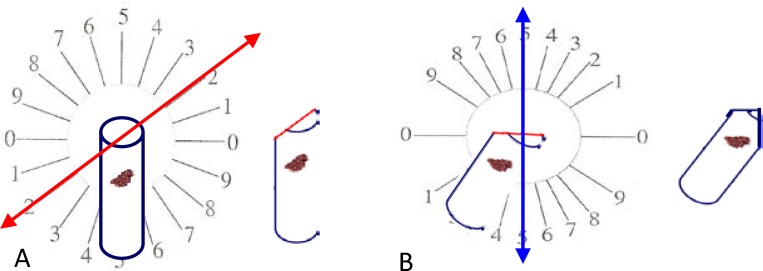
Orientator method: (A) The cylindrical paraffin block is placed on a φ clock. A random number between 0 and 9, for example 2 is selected and an appropriate cut was made along the selected number. (B).The block was then placed on the θ-clock along its cut surface on the 0-0 axis and a random number (for example 5) was selected and another cut was made along the selected number

Block was placed on the θ-clock along its cut surface on the 0-0 axis and a random number was selected and another cut was made along the selected number. Finally, 5 and 20 μm thick cuts were made using a microtome and stained with Hematoxylin and Eosin (H&E) method (Figure 1).

**The volume of ovary, cortex, medulla and corpus luteum (mm** ^3^ **)**

To estimate the mean total volume of the ovary, the images of 5 μm thick sections were transferred to the working table using the micro-projector (Neo Promar Leitz Germany) with 40× magnification. Then, the counting probe was randomly superimposed on the images, the points were counted and the total volume of the ovary was estimated using the Cavalieri methods applying the following formula (21): 

$V_{\mathrm{total ovary}}=\sum_{i=1}^{n}p\times a(p)\times t$

**Figure 2:**
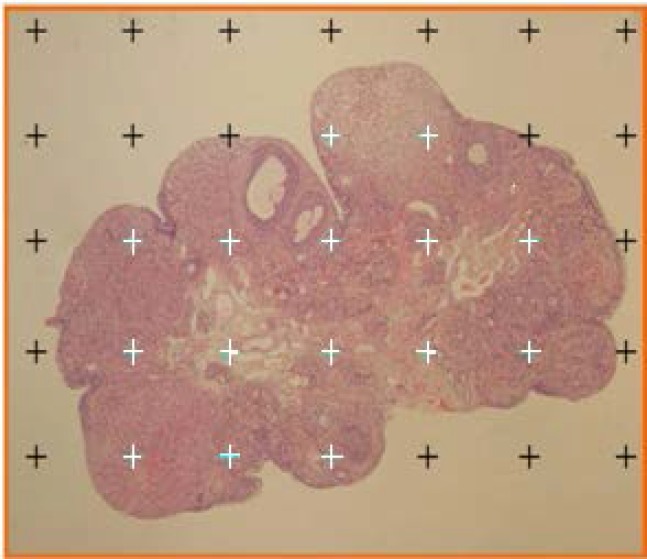
Estimating the ovary volume using the Cavalieri principle. Resulting points from the randomly superimposed probe on the images were counted (magnification ×400

Where Σ^n^_i=1_p is the total number of points superimposed on the image, (t) is the thickness of the section and a (p) is the area associated with each point (Figure 2). 

**The volume of oocyte and its nuclei (μm** ^3^ **)**

To estimate the oocyte volume , and their nuclei, the nucleator method was used. An average of 12 sections from 20 μm thick sections were randomly selected. Then, the selected follicles were studied using the Olympus microscope (BX 14 TE) with a 100× magnification. To estimate the volume of oocyte in the selected cells with an unbiased counting frame, the distance from the center of the nucleolus to the oocyte membrane was measured. To calculate the volume of oocyte nucleus the distance from the center of the nucleolus to the nucleus membrane was measured (Figure 4). The volume of each compartment was calculated using the following equation (21, 22, 25):

$V_{n}=4/3\pi \times \bar{L_{n}^{3}}$

L_n_: distance from the center of the nucleolus to the oocyte membrane.

**Zona pellucida thickness (μm)**

To estimate the mean thickness of zona pellucida, an average of 12 sections from 5 μm thick sections were randomly selected and studied using the Olympus microscope (BX 14 TE) with a 100× magnification. To determine measurement sites, the specific line grid (3 parallel lines) was randomly superimposed on the sampled fields (Figure 5). To calculate the mean thickness of the ZP, the orthogonal intercept method was used**.** The specific line grid (three parallel lines) was randomly superimposed on the sampled fields. The length of a perpendicular line extended from the inner membrane to the outer surface of zona pellucida at each intercept of the line of the grid with zona pellucida membrane was considered as the orthogonal intercept (oi). An average of 100-200 measurements was estimated and the harmonic mean thickness was calculated using the following formula (26):

$\mathrm{zona pellucidamean thickness}=\frac{8}{3\pi }\times \mathrm{number of measurments}/(\frac{1}{{\mathrm{oi}}_{1}}+\frac{1}{{\mathrm{oi}}_{2}}+\frac{1}{{\mathrm{oi}}_{3}}+\ldots )$

Orthogonal intercepts (oi): number of measurements

**Statistical analysis**

The results were analyzed by one-way ANOVA and Tukey's test, using the SPSS software version 16.00. p<0.05 were considered significant.

## Results

**Volume of ovary, cortex, medulla and corpus luteum (mm** ^3^ **)**

Mean total volume of ovary (p<0.01) and cortex volume (p<0.01) and medulla (p<0.04) showed a high significant reduction in BPA group compared to other groups, while co-administration of BPA with vitamin C increased the volume of ovary, cortex and medulla to the control level. The mean volume of corpus luteum reduced significantly in the BPA group compared to the vitamin C and BPA + vitamin C groups (p<0.01) (Table I).

**Number of follicles**

The mean number of antral follicles (p<0.001) reduced while the mean number of atretic follicles increased significantly (p<0.02) in the BPA group when compared to the other groups. In BPA + vitamin C, variations due to BPA exposure were compensated to the control level. Mean total number of primordial, primary and secondary follicles show no significant difference among various groups (Table II).

**Thickness of zona pellucida (μm)**

Comparing the mean thickness of zona pellucida in secondary (p<0.02) and antral follicles (p<0.01) showed a significant reduction in BPA group, while co-treatment of rats with BPA + vitamin C compensated these reductions to the control level (Table III).

**Volume of oocyte (μm** ^3^ **)**

Mean volume of oocyte in antral follicles reduced significantly in BPA group compared to other groups (p<0.004), while this reduction was compensated in BPA + vitamin C and vitamin C groups to control level. The mean volume of oocyte in primordial, primary and secondary follicles showed no significant difference in any groups (Table IV). 

**Volume of oocyte nucleus (μm** ^3^ **)**

Mean volume of oocyte nucleus (μm^3^) in antral follicles showed highly significant reduction in BPA group compared to other groups (p<0.001). Meanwhile the mean volume of oocyte nucleus in antral follicles increased significantly in BPA + vitamin C group. The volume of oocyte nucleus of primordial, primary and secondary follicles showed no significant difference between all groups (Table V).

**Body and ovary weight (g)**

Mean body weight (p>0.52) of rats and also the ovary weight (p>0.44) showed no significant difference in groups at the end of treatment (Table VI).

**Histopathological findings**

The structure of the ovarian tissue was found to be normal in control and vitamin C groups. Several ovary cysts were observed in BPA group. Meanwhile, there was no significant variation in the ovary tissue of BPA + vitamin C group compared to control (Figure 6).

**Table I:** Comparing the mean total volume of ovary, cortex, medulla and corpus luteum (mm^3^) in different groups of rats 20 days after treatment with BPA and vitamin C. Means with different code letters in each column differ significantly from each other

**Groups (n=6)**	**Volume of ovary (mm** ^3^ **)**	**Volume of cortex (mm** ^3^ **)**	**Volume of medulla (mm** ^3^ **)**	**Volume of corpus luteum (mm** ^3^ **)**
Control	18.16 ± 2.23^a^	15.77 ± 1.66^a^	2.39 ± 0.45^a^	7.03 ± 1.32^a^
BPA	13.18 ± 0.89^b^	11.73 ± 0.79^b^	1.45 ± 0.28^b^	4.81 ± 0.49^b^
V C	17.42 ± 2.41^a^	14.96 ± 1.80^a^	2.46 ± 0.51^a^	8.22 ± 0.92^a^
BPA+Vitamin C	17.03 ± 1.63^a^	14.67 ± 1.34^a^	2.36 ± 0.47^a^	6.94 ± 0.59^a^
p-value	0.01^*^	0.01^*^	0.04^*^	0.01^*^

**Table II:** Comparing the mean the number of primordial, primary, secondary, antral, and atretic follicles in different groups of rats 20 days after treatment with BPA and vitamin C. Means with different code letters in each column differ significantly from each other

**Groups (n=6)**	**Follicles**				
	**Primordial**	**Primary**	**Secondary**	**Antral**	**Atretic**
Control	3164 ± 219.49^a^	1235 ± 117.2^a^	488 ± 86.38^a^	247 ± 20.97^a^	61 ± 7.5^a^
BPA	2957 ± 265.35^a^	942 ± 150.69^a^	459 ± 90.50^a^	153 ± 13.89^b^	80 ± 8.9^b^
V C	3593 ± 239.98^a^	1159 ± 141.10^a^	519 ± 75.45^a^	302 ± 66.69^a^	57 ± 5.7^a^
BPA+Vitamin C	3241 ± 237.54^a^	1256 ± 118.12^a^	510 ± 58.39^a^	277 ± 31.50^a^	60 ± 8.7^a^
p-value	0.63	0.98	0.83	0.001^*^	0.02^*^

**Table III:** Comparing the mean thickness of ZP (μm) in the antral and secondary follicles in different groups of rats 20 days after treatment with BPA and vitamin C. Means with different code letters in each column differ significantly from each other

**Groups (n=6)**	**ZP thickness(µm)**	
	**Secondary follicles**	**Antral follicles**
Control	14.31 ± 1.08^a^	18.08 ± 0.72^a^
BPA	12.75 ± 0.36^b^	15.76 ± 0.50^b^
V C	14.24 ± 0.80^a^	18.85 ± 0.46^a^
BPA+Vitamin C	14.96 ± 0.7^a^	18.01 ± 0.63^a^
p-value	0.02^*^	0.01^*^

**Table IV:** Comparing the mean volume of oocyte (μm^3^) in different types of follicles in different groups of rats 20 days after treatment with BPA and vitamin C. Means with different code letters in each column differ significantly from each other

**Groups (n=6)**	**Oocyte volume (µm** ^3^ **)**			
	**Primordial**	**Primary**	**Secondary**	**Antral**
Control	1335.93 ± 92.1^a^	3782.08 ± 341.9^a^	47599.44 ± 3346.2^a^	144948.01 ± 10484.7^a^
BPA	1285.24 ± 102.1^a^	3599.61 ± 392./7^a^	41986.33 ± 4168.7^a^	113752.92 ± 14781.3^b^
V C	1366.70 ± 30.9^a^	3885.00 ± 298.5^a^	57775.24 ± 2074.0^a^	148267.00 ± 11478.1^a^
BPA+Vitamin C	1337.43 ± 80.2^a^	3650.09 ± 317.5^a^	45857.86 ± 3180.5^a^	145947.73 ± 15690.1^a^
p-value	0.51	0.41	0.58	0.004^*^

**Table V:** Comparing the mean volume of oocyte nucleus (μm^3^) in different types of follicles in the groups of rats 20 days after treatment with BPA and vitamin C. Means with different code letters in each column differ significantly from each other

**Groups (n=6)**	**Oocyte nucleus volume (µm** ^3^ **)**		
	**Primary follicles**	**Secondary follicles**	**Antral follicles**
Control	655.19 ± 57.36^a^	3263.67 ± 233.32^a^	5114 ± 378.69^a^
BPA	615.05 ± 61.45^a^	3069.13 ± 145.62^a^	4008.80 ± 123.87^b^
V C	618.34 ± 25.34^a^	4236.71 ± 217.82^a^	5395.63 ± 361.55^a^
BPA+Vitamin C	599.52 ± 53.29^a^	3440.92 ± 342.51^a^	5342.63 ± 297.01^a^
p-value	0.31	0.11	0.001*

**Table VI:** Comparing the mean body and ovary weight (g) in different groups of rats 20 days after treatment with BPA and vitamin C. Means with different code letters in each column differ significantly from each other

**Groups (n=6)**	**Body Weight (gr)** **(at the start of treatment)**	**Body Weight (gr)** **(at the end of treatment)**	**Weight of ovary (gr)**
Control	207.16 ± 13.84^a^	210.33 ± 15.50^a^	0.037 ± 0.003^a^
BPA	208.50 ± 14.70^a^	211.16 ± 16.63^a^	0.035 ± 0.004^a^
V C	203.83 ± 10.43^a^	200.83 ± 13.46^a^	0.032 ± 0.008^a^
BPA+Vitamin C	204.00 ± 13.76^a^	201.83 ± 15.30^a^	0.036 ± 0.007^a^
p-value	0.90	0.52	0.44

**Figure 3:**
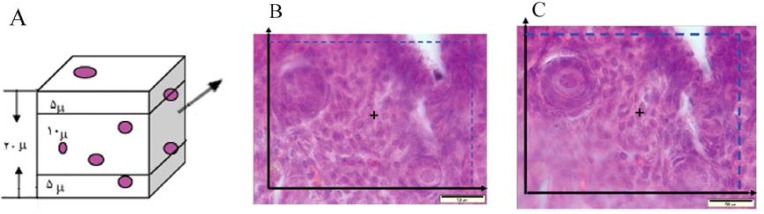
Estimating the number of follicles using the optical disector. A. 5 µm from the top and bottom of the sections was ignored as the guard area against the cutting artifacts. B, C. An unbiased counting frame superimposed on the selected field was used to sample the nucleoli profiles of the oocytes (magnification ×1000

**Figure 4:**
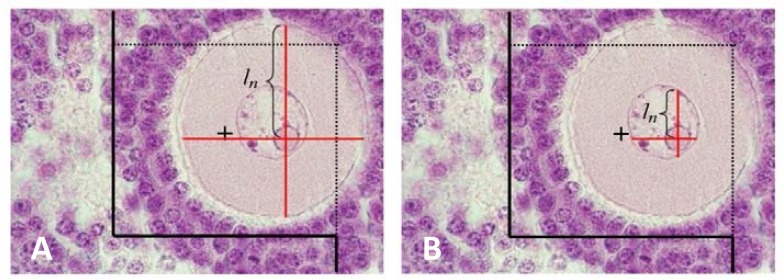
Estimating the mean volumes of oocytes and their nuclei by using the unbiased stereological technique of the nucleator. For each sampled oocyte, an isotropic direction is generated from a random point within the nucleolus, and the distances in each direction out from the point to the boundary of the oocyte membrane (A) (to estimate the oocyte volume), and the nucleus (B) (to estimate the nuclear volume) are considered (magnification ×1000

**Figure 5:**
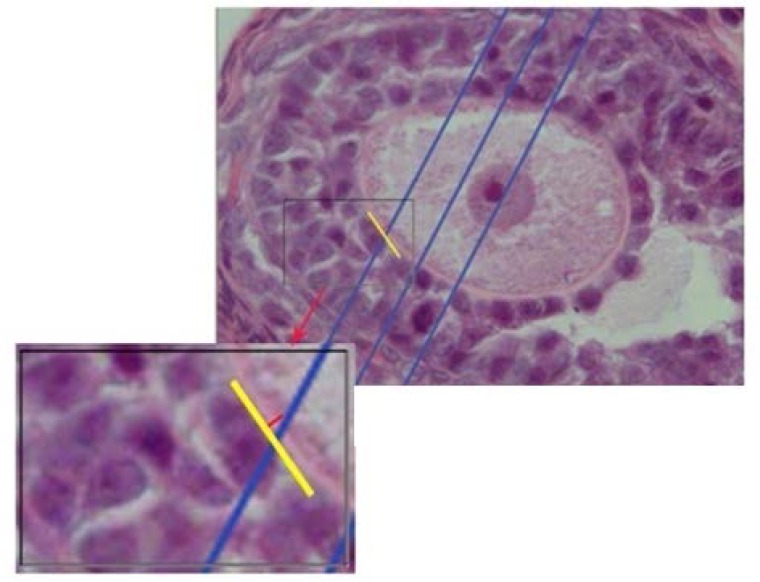
Estimating the mean thickness of zona pellucida using orthogonal intercept method. (magnification ×1000

**Figure 6:**
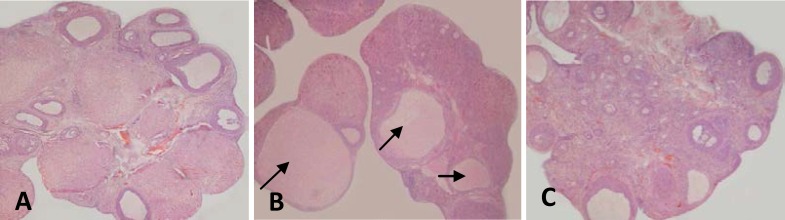
Micrographs of the ovary tissue in rats treated with BPA and vitamin C (H&E staining and 40 x magnification) representing the histopathologic variations of the ovarian tissue: (A) Control group representing the normal structure of the ovary tissue (B) BPA group showing an increase in the number of ovarian cysts indicated by ↗ (C) BPA+ vitamin C group showing the normal structure of the ovary

## Discussion

The results showed a reduction in total volume of ovary, cortex, medulla and corpus luteum in rats treated with BPA. According to Fernandez* et al*, treatment of female rats with BPA (50 µg/50 µL) for a period of 10 days postnatal causes ovary atrophy, an increase in the number of atretic follicles and a reduction in the number of antral follicles and corpus luteum which leads to cortex and ovary volume reduction. BPA can reduce the estradiol level and consequently the medullary cells number (12, 27, 28). On the other hand, it causes apoptosis and shrinkage in granulosa cells which could explain why BPA exposure decreases the ovary volume (29).

Since BPA releases free radicals, it can also cause a reduction in the ovary volume through follicles atresia and ovary apoptosis as a result of oxidative stress (10, 11, 30). Other investigations also showed that BPA causes follicular atresia and induces apoptosis in granulosa cells (12, 31). In addition, our investigation indicated a significant reduction in the mean thickness of zona pellucida, a key factor in oogenesis, fertilization, and preimplantation development, in BPA treated group. Since oocyte and follicular cells both participate in the formation of zona pellucid therefore follicular atresia and the apoptosis of granulosa cells could be the underlying cause for the reduction in ZP thickness (32, 33). Furthermore, the production of free radicals and lipid peroxidation following BPA exposure can degrade the lipid and protein components of ZP, which could be considered as another reason for observed reduction in ZP thickness (10, 11, 34). 

As mentioned above, in this study we also observed that the mean volume of oocyte and its nucleus in antral follicles decreased significantly in BPA group. This could be due to the fact that BPA exposure causes a reduction in the levels of LH and estradiol hormones, inhibiting the growth of antral follicles and cell cycle progression in the oocyte and inducing apoptosis in the granulosa cells (2, 27, 31, 35). These factors can affect oocyte development leading to a reduction in the mean volume of oocyte and its nucleus.

Treatment with BPA also caused a reduction in the number of antral follicles and an increase in the number of atretic follicles which is in agreement with Fernández *et al* results (12). The investigation by Gupta *et al* also showed that methoxychlor causes follicular atresia and a reduction in the number of antral follicles through inducing oxidative stress (37). Since BPA is considered as an inducer of oxidative stress, therefore treatment with BPA can also cause follicular degeneration and an increase in the number of atretic follicles (10, 11). Estradiol is responsible for increasing the number of antral follicles and helping their maturation and since BPA causes a reduction in estradiol, it can be deduced that BPA can affect the number of antral follicles (27, 36). 

Co-administration of BPA with vitamin C compensated for the reduction in volume of ovary, cortex, medulla, corpus luteum and volume of oocyte and its nucleus in antral follicles to the control level. The mean thickness of zona pellucida and the mean number of antral follicles were also compensated to the control level following treatment with BPA and vitamin C. 

This can be due to the function of vitamin C in synthesizing the components of extracellular matrix especially hydroxylation of collagen (38). Vitamin C can also stimulate the secretion of gonadotrophins which was reduced as a consequence of BPA treatment (16). It should be noted that vitamin C as a strong antioxidant can prevent the oxidative stress induced by other toxicants such as hexavalent chromium and sodium arsenite (16, 17). Chitra *et al* also reported that co-treatment of vitamin C and BPA compensates the effects of oxidative stress in the sperm and epididym of rats (39). Therefore, vitamin C can compensate for the undesired effects of BPA through the prevention of oxidative stress caused by BPA. In addition, vitamin C can clean away free radicals and protect proteins against their harmful effect, therefore preventing the loss of proteins and the reduction in the thickness of zona pellucida and volume of the oocyte and its nucleus (40).

In our study, the ovarian weights in the adult rats did not significantly change following BPA exposure which is in accordance with the results of other studies (7, 41). However it should be mentioned that some studies have shown that increasing the dose of BPA or infantile exposure to PBA may lead to a reduction in the ovary weight therefore it could be deduced that the dose of BPA used or the time of exposure may influence the changes observed in the ovary weight (42). There was also no significant difference in the rats body weight among the groups which is similar to the results obtained by other studies (7, 14, 43). 

Although some studies have concluded that post natal and prepuberty exposures to BPA may affect the body weight in a dose dependant manner but some studies have considered the toxic effect of BPA on the body weight in adult rats insignificant (7, 44). Therefore, due to the variety of exposure methods and doses, definitive conclusions on the effect of BPA on body weight is hard to gain (45, 46).

## Conclusion

We conclude that vitamin C, through its antioxidant activity, can compensate for the adverse effects of BPA treatment on the volume of ovary, cortex, medulla, corpus luteum and also the number of antral follicles and the mean thickness of zona pellucida in rats. Therefore, the consumption of vitamin C can be useful where exposure to environmental toxicants such as BPA in cities and industrial centers are inevitable.
